# Knowledge, attitude, and practice of cardiovascular disease prevention among patients with type 2 diabetes and/or overweight or obesity

**DOI:** 10.3389/fpubh.2025.1712899

**Published:** 2025-11-27

**Authors:** Xiangming Zhou, Chunlan Zou, Xin Li, Hongxi Tian, Yuanyuan Miao, Tingyu Ke, Ling Zhao

**Affiliations:** Department of Endocrinology, The 2nd Affiliated Hospital of Kunming Medical University, Kunming, Yunnan, China

**Keywords:** knowledge, attitude, health behavior, cardiovascular diseases/prevention & control, diabetes mellitus, type 2, obesity, cross-sectional studies

## Abstract

**Introduction:**

Cardiovascular diseases (CVDs), encompassing various disorders of the heart and blood vessels, remain a major challenge to global health. From 1990 to 2019, the number of people suffering from CVDs worldwide surged from 270 million to 523 million. This study aimed to investigate the levels of knowledge, attitude, and practice (KAP) toward cardiovascular disease prevention among patients with type 2 diabetes and/or overweight or obesity.

**Methods:**

This cross-sectional study was conducted between January and July, 2024, at the Second Affiliated Hospital of Kunming Medical University, among voluntary patients with T2D and/or overweight or obesity, using a validated, self-designed questionnaire. A total of 932 (93.11%) valid questionnaire responses were analyzed. Among the participants, 52.79% were male, with a mean age of 49.76 ± 17.24 years.

**Results:**

The mean scores of KAP were 15.34 ± 6.10 (possible range: 0–26), 39.66 ± 3.69 (possible range: 11–55), and 41.38 ± 7.57 (possible range: 12–60), respectively. Path analysis indicated that knowledge had both direct (*β* (95% CI) = 0.308 (0.234 ~ 0.384), *p* = 0.005) and indirect (β (95% CI) = 0.081 (0.053 ~ 0.118), *p* = 0.006) effects on practice.

**Conclusion:**

Patients with type 2 diabetes and/or overweight or obesity exhibited a positive attitude but demonstrated moderate levels of knowledge and practice, indicating that while awareness exists, further strategies are needed to enhance effective cardiovascular disease prevention behaviors.

## Introduction

Cardiovascular diseases (CVDs), encompassing various disorders of the heart and blood vessels, remain a major challenge to global health. From 1990 to 2019, the number of people suffering from CVDs worldwide surged from 270 million to 523 million (1). According to the report released by American Heart Association (AHA), CVDs caused approximately 19.91 million deaths worldwide in 2021 (2). These diseases, including coronary heart disease, stroke, heart failure, and peripheral vascular disease, are often attributed to a complex interplay of modifiable and non-modifiable risk factors (3). Among the modifiable risk factors, type 2 diabetes mellitus (T2D) and overweight or obesity stand out as major contributors to the development and progression of CVDs (4). The prevalence of these conditions has been escalating globally, paralleled by an alarming rise in CVD incidence, highlighting the urgent need for effective risk factor management strategies (5).

The relationship between T2D and/or overweight/obesity and CVDs is well established. Patients with T2D are at a heightened risk of developing CVD due to hyperglycemia-induced endothelial dysfunction, inflammation, and dyslipidemia (6, 7). Similarly, overweight or obese individuals experience metabolic alterations, including insulin resistance, hypertension, and dyslipidemia, all of which contribute to the development of CVD (8, 9). Consequently, effectively managing cardiovascular risk factors in this patient group is essential for alleviating the overall impact of CVDs.

Knowledge, Attitude, and Practice (KAP) studies represent a structured survey methodology that has been widely employed in health research to assess individuals’ understanding of a health issue, their beliefs and attitudes toward it, and their actual behaviors related to it (10). In the context of CVD risk factor management among patients with T2D and/or overweight/obesity, KAP method is able to provide valuable insights into the gaps in knowledge, misconceptions, and barriers that may hinder effective risk reduction efforts. Yang et al. have investigated the KAP regarding CVD prevention among middle school students in China (11). Siddique et al. have organized a face-to-face interview to explore the KAP regarding CVDs among older individuals of rural Bangladesh (12). Verma et al. investigated the knowledge, attitude, and practices (KAP) related to health behaviors and cardiovascular risk factors among patients with metabolic syndrome at a teaching hospital in India (13). As individuals belonging to the high-risk demographic for cardiovascular diseases, diabetic patients and obese individuals require a heightened focus on managing their cardiovascular risk compared to the general healthy population.

Patients with type 2 diabetes and those with overweight or obesity often present overlapping metabolic abnormalities, such as insulin resistance, dyslipidemia, and systemic inflammation, which synergistically increase cardiovascular risk. Previous studies have predominantly investigated these populations separately or focused on general or healthy individuals, without assessing their combined high-risk characteristics (14–16). Moreover, no existing study has evaluated the knowledge, attitude, and practice regarding cardiovascular disease prevention specifically in patients who have either or both of these conditions. Therefore, this study aims to fill this gap by investigating the KAP toward cardiovascular disease prevention among individuals with type 2 diabetes and/or overweight or obesity.

## Materials and methods

### Study design and participants

This cross-sectional research was carried out from January 5 to July 10, 2024, at the Second Affiliated Hospital of Kunming Medical University. A random selection of patients who had T2D and/or overweight or obesity were included. The inclusion criteria were as follows: (1) Patients with T2D; (2) Overweight individuals; (3) Obese individuals. Overweight and obesity are classified using the Body Mass Index (BMI), determined by the formula: BMI = weight (kg) / height^2^ (m^2^). A BMI ranging from 25 to 29.9 indicates overweight status, whereas a BMI of 30 or higher is categorized as obesity (17). The exclusion criteria were as follows: (1) Individuals unwilling to participate; (2) Individuals under 18 years old. The study was approved by the Medical Ethics Committee of the Second Affiliated Hospital of Kunming Medical University(S-PJ-K-2023-313), and written informed consent was obtained from all participants. A convenience sampling method was employed to recruit eligible participants who attended the endocrinology outpatient clinic during the study period.

### Questionnaire design

The questionnaire was independently developed with reference to the *Comprehensive Management of Cardiovascular Risk Factors for Adults with Type 2 Diabetes* by the AHA (18) and *Obesity and cardiovascular disease: mechanistic insights and management strategies* from the World Heart Federation and World Obesity Federation (19). The questionnaire was then modified based on feedback from three experts. Prior to its administration, it underwent pre-testing with a sample size of 35 participants, resulting in a total Cronbach’s *α* coefficient of 0.839, which reflects strong internal reliability. Additionally, confirmatory factor analysis (CFA) was performed to evaluate construct validity. The model fit indices demonstrated an acceptable fit (RMSEA = 0.052, IFI = 0.923, TLI = 0.916, CFI = 0.923), supporting the validity of the questionnaire.

The finalized questionnaire, originally in Chinese (with an English-translated version provided in the Appendix), includes a total of 53 items distributed across four sections: basic information (17 items), knowledge (13 items), attitudes (11 items), and practices (12 items). Specifically, the knowledge section is scored on a scale from 0 to 26, with responses rated as follows: “very familiar” earns 2 points, “heard of it” receives 1 point, and “not clear” is given 0 points. Meanwhile, both the attitude and practice dimensions utilize a five-point Likert scale, spanning from very positive (5 points) to very negative (1 point). Specifically, for the attitude dimension, items 1/3/4/5/6/7/9/10/11 are scored in descending order (a = 5, b = 4, c = 3, d = 2, e = 1), whereas item 2/8 are scored in ascending order (a = 1, b = 2, c = 3, d = 4, e = 5), yielding a potential score range of 11 to 55. Furthermore, in practice dimension, except for items 4 and 5, which are scored in ascending order (a = 1, b = 2, c = 3, d = 4, e = 5), other items are scored in descending order (a = 5, b = 4, c = 3, d = 2, e = 1). The score range is likely to be between 12 and 60.

After completing the questionnaire, average scores for the knowledge dimension categorize respondents into three levels: insufficient knowledge (0–13 points), moderate knowledge (14–18 points), and sufficient knowledge (19–26 points). Similarly, average scores for the attitude dimension distinguish between negative (11–27 points), neutral (28–38 points), and positive attitudes (39–55 points). Lastly, average scores for the practice dimension classify individuals into negative (12–30 points), moderate (31–42 points), and positive practice behaviors (43–60 points).

### Questionnaire distribution and quality control

The questionnaire was administered to individuals with T2D and/or overweight or obesity via Wenjuanxing on the WeChat Mini Program, using a QR code for distribution and data collection. Participants accessed and filled out the survey by scanning the QR code with WeChat. To maintain data integrity and completeness, each IP address was limited to a single submission, and all questions were required to be answered. The research team examined all submitted questionnaires to ensure they were complete, logically consistent, and reasonable in content. To control for quality, responses were considered invalid if the completion time was < 90 s or if any KAP section had all responses selected as the same option. In addition, the Cronbach’s *α* coefficient for the overall valid questionnaire was 0.899, indicating good internal consistency.

### Sample size

According to the formula for calculating the sample size in cross-sectional surveys: $n={\left(\frac{Z_{1-\alpha /2}}{\delta }\right)}^{2}\times p\times (1-p)$ In the formula, “*n*” represents the sample size for each group, “*α*” represents the type I error, which is typically set at 0.05, $Z_{1-\alpha /2}$ = 1.96, *δ* represents the allowable error, typically set at 0.05, and “*p*” is set at 0.5 (as setting it at 0.5 maximizes the value and ensures a sufficiently large sample size). The calculated sample size “*n*” is 384. Considering an estimated questionnaire response rate of 80%, the final plan is to collect 480 valid questionnaires.

### Statistical analysis

Statistical analysis was performed using SPSS 26.0 (IBM Corp., Armonk, N. Y., USA). Quantitative data were described using Mean ± SD, and group comparisons were conducted using ANOVA. Categorical data were described using frequencies (percentages). Pre- and post-comparisons for knowledge and attitude used chi-square / Fisher’s exact tests. The correlation between knowledge, attitude, and willingness scores was analyzed using Spearman’s rank correlation. Structural Equation Modeling (SEM) with path analysis and mediation analysis was used to explore the pathways between demographic data and KAP, as well as among the three KAP components. All statistical tests were two-tailed, with a *p*-value of <0.05 considered statistically significant.

## Results

### Participant demographics and baseline characteristics

A total of 932 valid responses were included in the final analysis, representing a clinically meaningful sample of individuals at elevated cardiovascular risk. Detailed demographic and clinical characteristics, including age distribution, sex, education level, and disease categories, are presented in Table 1.

**Table 1:** Baseline characteristics of patients and KAP scores.

Variables	*N* (%)/Mean ± SD	Knowledge Mean ± SD	*p*	Attitude Mean ± SD	*p*	Practice Mean ± SD	*p*
Total score		15.34 ± 6.10		39.66 ± 3.69		41.38 ± 7.57	
Age	49.76 ± 17.24						
Gender			0.563		0.778		**< 0.001**
Male	492 (52.79)	15.22 ± 6.43		39.68 ± 3.68		40.32 ± 7.95	
Female	440 (47.21)	15.47 ± 5.72		39.63 ± 3.71		42.56 ± 6.93	
Ethnicity			0.079		**0.047**		**0.036**
Han	752 (80.69)	15.18 ± 6.19		39.54 ± 3.60		41.14 ± 7.48	
Minority	180 (19.31)	15.99 ± 5.66		40.17 ± 4.02		42.37 ± 7.86	
Place of residence			**0.011**		0.089		**< 0.001**
Provincial capital city	594 (63.73)	15.36 ± 6.03		39.54 ± 3.71		42.00 ± 7.76	
Non-provincial capital city	85 (9.12)	16.29 ± 6.06		40.02 ± 3.94		42.04 ± 6.53	
County	129 (13.84)	16.16 ± 5.94		40.27 ± 3.82		40.67 ± 6.78	
Township/Village	124 (13.3)	13.74 ± 6.37		39.34 ± 3.23		38.72 ± 7.49	
Education level			**< 0.001**		**< 0.001**		**0.035**
Middle school or below	317 (34.01)	12.30 ± 5.53		38.49 ± 3.16		40.32 ± 7.29	
High school/technical school/associate’s degree	303 (32.51)	15.81 ± 5.64		39.64 ± 3.46		41.89 ± 7.66	
Bachelor’s degree	274 (29.4)	17.55 ± 5.63		40.70 ± 3.89		41.77 ± 7.62	
Master’s degree and above	38 (4.08)	21.05 ± 5.49		42.05 ± 4.74		43.39 ± 7.87	
Current employment status			**< 0.001**		**< 0.001**		**< 0.001**
Employed	353 (37.88)	17.47 ± 5.88		40.41 ± 3.84		41.14 ± 7.92	
Unemployed	48 (5.15)	13.77 ± 4.89		38.50 ± 3.03		39.35 ± 8.26	
Retired	303 (32.51)	14.57 ± 5.64		39.10 ± 3.41		43.04 ± 6.59	
Other	228 (24.46)	13.39 ± 6.29		39.27 ± 3.77		39.96 ± 7.68	
Household’s monthly per capita income			**< 0.001**		**< 0.001**		**< 0.001**
< 2000	147 (15.77)	11.84 ± 6.51		38.62 ± 3.16		38.77 ± 8.00	
2000–5,000	400 (42.92)	15.16 ± 5.48		39.42 ± 3.50		41.45 ± 7.29	
5,000–10,000	260 (27.9)	16.28 ± 6.02		39.93 ± 3.60		42.29 ± 7.25	
10,000–20,000	77 (8.26)	18.30 ± 5.54		41.21 ± 4.22		42.19 ± 7.03	
> 20,000	48 (5.15)	17.75 ± 6.12		40.83 ± 5.01		42.52 ± 9.23	
Marital status			**< 0.001**		**0.002**		**0.040**
Single	158 (16.95)	16.82 ± 6.03		40.48 ± 4.16		40.04 ± 8.46	
Married	692 (74.25)	15.17 ± 6.08		39.55 ± 3.54		41.69 ± 7.31	
Divorced	27 (2.9)	15.30 ± 5.74		40.19 ± 3.44		40.37 ± 9.52	
Widowed	55 (5.9)	13.22 ± 5.99		38.40 ± 3.78		41.76 ± 6.63	
Any of the following conditions			**< 0.001**		**< 0.001**		**< 0.001**
Type 2 diabetes	427 (45.82)	14.81 ± 5.75		39.34 ± 3.53		43.24 ± 6.92	
Overweight	208 (22.32)	17.14 ± 6.24		40.58 ± 3.76		40.15 ± 7.45	
Obesity	108 (11.59)	15.30 ± 6.12		39.29 ± 4.00		37.63 ± 7.99	
Type 2 diabetes with overweight	129 (13.84)	14.36 ± 6.25		39.63 ± 3.58		40.68 ± 7.52	
Type 2 diabetes with obesity	60 (6.44)	15.02 ± 6.55		39.47 ± 3.85		40.63 ± 8.18	
Duration of type 2 diabetes			< 0.001		0.008		< 0.001
< 1 year	125 (13.41)	13.16 ± 5.89		39.70 ± 3.85		40.64 ± 9.21	
≤ 1 years ≥ 3 years	96 (10.3)	15.17 ± 5.61		39.56 ± 3.45		44.35 ± 6.09	
≥ 3 years	374 (40.13)	15.11 ± 5.94		39.23 ± 3.45		42.61 ± 6.68	
Duration of overweight/obesity			< 0.001		0.118		< 0.001
< 1 year	76 (8.15)	15.57 ± 6.20		39.76 ± 4.24		40.45 ± 7.62	
≥ 1 years ≤ 3 years	117 (12.55)	17.48 ± 5.81		40.22 ± 3.89		40.67 ± 8.01	
≥ 3 years	261 (28)	15.15 ± 6.54		39.91 ± 3.59		39.26 ± 7.83	
Current treatment methods:			**< 0.001**		**< 0.001**		**< 0.001**
Lifestyle modification	253 (27.15)	16.89 ± 5.91		40.61 ± 3.96		41.24 ± 7.42	
Medication/surgical treatment	538 (57.73)	15.02 ± 5.95		39.32 ± 3.39		42.94 ± 6.83	
No intervention	141 (15.13)	13.77 ± 6.46		39.26 ± 4.00		35.67 ± 7.79	
Using weight loss and metabolic medications			**< 0.001**		0.597		**0.004**
Yes	86 (9.23)	18.47 ± 6.09		39.84 ± 3.44		43.66 ± 6.64	
No	846 (90.77)	15.02 ± 6.01		39.64 ± 3.72		41.15 ± 7.62	
Health insurance type			**< 0.001**		**0.049**		0.130
Only social health insurance	763 (81.87)	14.87 ± 6.07		39.55 ± 3.51		41.13 ± 7.34	
Only commercial health insurance	13 (1.39)	19.00 ± 7.07		41.38 ± 3.33		43.54 ± 9.11	
Both social health insurance and commercial health insurance	127 (13.63)	17.81 ± 5.55		40.24 ± 4.63		43.06 ± 8.06	
No insurance	29 (3.11)	15.21 ± 6.01		39.34 ± 3.70		39.69 ± 9.59	
Self-assessed health status			**< 0.001**		**0.011**		**0.008**
Excellent	64 (6.87)	19.03 ± 6.07		41.17 ± 5.77		43.31 ± 10.05	
Good	270 (28.97)	15.49 ± 5.97		39.56 ± 3.62		41.93 ± 7.34	
Fair	455 (48.82)	15.18 ± 6.14		38.59 ± 3.49		41.36 ± 7.13	
Poor/very poor	143 (15.34)	13.90 ± 5.61		39.39 ± 3.11		39.53 ± 7.76	
Self-assessed life satisfaction			**< 0.001**		**< 0.001**		**< 0.001**
Very satisfied	91 (9.76)	18.07 ± 5.59		41.62 ± 4.42		43.91 ± 8.53	
Satisfied	496 (53.22)	15.64 ± 6.11		39.80 ± 3.47		42.06 ± 7.08	
Neutral	309 (33.15)	14.27 ± 5.93		39.02 ± 3.47		40.08 ± 7.55	
Dissatisfied/very dissatisfied	36 (3.86)	13.47 ± 6.12		38.31 ± 4.56		36.72 ± 8.04	

SD, standard deviation. Bold indicates a statistically significant difference.

### Knowledge, attitude and practice

The average scores of knowledge, attitude and practice dimensions were 15.34 ± 6.10 (possible range: 0–26), 39.66 ± 3.69 (possible range: 11–55), and 41.38 ± 7.57 (possible range: 12–60), respectively. The participants exhibited a positive attitude, but moderate knowledge and practice level regarding cardiovascular risk factor management. Generally, education level, employment status, income, marital status, having T2D / overweight / obesity and time duration of having these conditions, treatment methods, self-assessed health status and self-assessed life satisfaction all exerted significant influences on the scores across all three dimensions of KAP (all *p* < 0.05) (Table 1).

Within the knowledge dimension, the item regarding the dangers of unhealthy lifestyle habits, including smoking and heavy alcohol use, received the highest score, with 42.92% of participants selecting “very familiar” (K4). Furthermore, the item about benefit of moderate physical exercise and activity in maintaining cardiovascular health also got high accuracy, with 42.17% choosing “very familiar” (K3). While the lowest score was observed for the item about the benefit of healthy foods and the Mediterranean diet, with 23.18% choosing “very familiar” (K2). This might be due to the limited understanding of the Mediterranean diet among participants (Supplementary Table S1).

In the attitude dimension, the item emphasizing the significance of routine health examinations received the highest score, with 33.8% of respondents selecting “strongly agree” (A1). However, only 1.5% of the participants expressed absolute lack of concern for their cardiovascular health, marking the lowest score among all surveyed aspects (A2). This indicates a lack of confidence among the participants regarding their ability to prevent and manage cardiovascular diseases effectively. Besides, only 2.36% were strongly disagree with taking medication rather than lifestyle adjustments (A8) (Supplementary Table S2).

Within the practice dimension, the item related to smoking achieved the highest score, with 59.12% of participants indicating “Never” (P5). While the lowest score was observed for the item about consulting professionals to improve diet and lifestyle, with only 9.55% choosing “Always” (P12). Besides, the support rates for regularly monitoring blood parameters (P1), controlling dietary intake (P2), exercising (P8), actively seeking relevant information (P9), regularly communicating with doctors (P10), and participating in relevant educational guidance (P11) were also relatively low, with only 11.59, 12.88, 12.23, 12.12, 11.16, and 10.41% of participants choosing “Always” respectively (Supplementary Table S3).

### Correlation analysis

The findings showed strong positive correlations between knowledge and attitude (*r* = 0.456, *p* < 0.001), knowledge and practice (*r* = 0.351, *p* < 0.001), and attitude and practice (*r* = 0.286, *p* < 0.001) (Table 2).

**Table 2:** Correlation analysis.

Dimension	Knowledge	Attitude	Practice
Knowledge	1		
Attitude	0.456 (*p* < 0.001)	1	
Practice	0.351 (*p* < 0.001)	0.286 (*p* < 0.001)	1

### Path analysis

**Figure 1:**
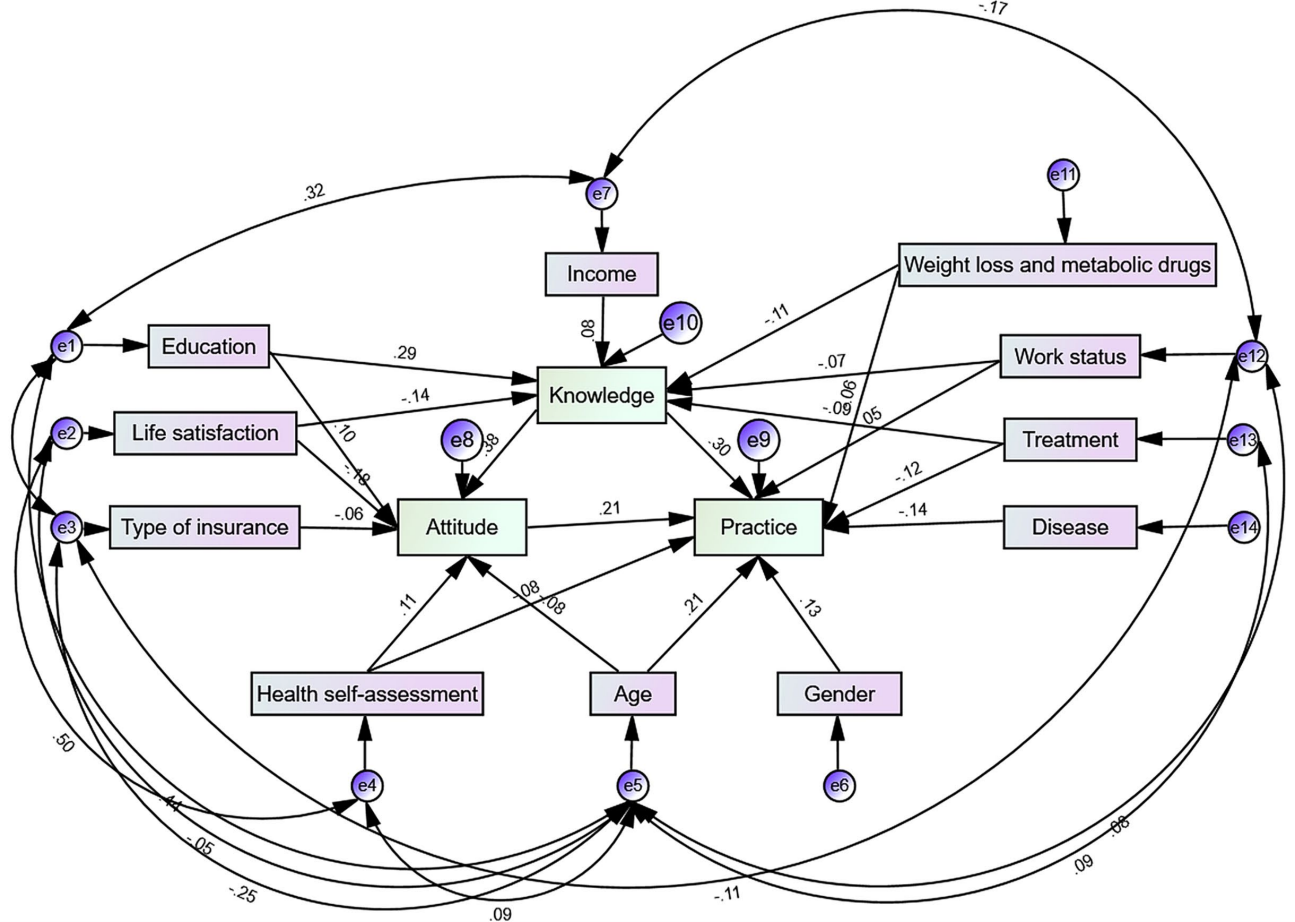
Path analysis diagram.

Initially, variables that exhibited significant differences in their effects on KAP scores within the basic information (Table 1) were incorporated into the SEM framework for path analysis (Supplementary Tables S4, S5). Subsequently, variables that showed no statistical significance (*p* > 0.05) in the path analysis were removed, resulting in a streamlined SEM analysis (Figure 1). All model fitting parameters met the established criteria (Supplementary Table S6), with the total effects estimate presented in Supplementary Table S7.

Firstly, taking weight loss and metabolic drugs (*β* (95% confidence interval (CI)) = −0.108 (−0.164 ~ −0.050), *p* = 0.009), life satisfaction (*β* (95% CI) = −0.136 (−0.202 ~ −0.077), *p* = 0.007), treatment (*β* (95% CI) = −0.094 (−0.151 ~ −0.032), *p* = 0.019), work status (*β* (95% CI) = −0.072 (−0.139 ~ −0.003), *p* = 0.042) and education (*β* (95% CI) = 0.280 (0.206 ~ 0.357), *p* = 0.010) all had direct effects on knowledge (Table 3).

**Table 3:** Path analysis after removing paths with *p* > 0.05.

Model paths		Standardized total effects		Standardized direct effects		Standardized indirect effects	
		β (95% CI)	*p*	β (95% CI)	*p*	β (95% CI)	*p*
Knowledge	←						
	Weight loss and metabolic drugs	−0.108 (−0.164 ~ −0.050)	0.009	−0.108 (−0.164 ~ −0.050)	0.009		
	Life satisfaction	−0.136 (−0.202 ~ −0.077)	0.007	−0.136 (−0.202 ~ −0.077)	0.007		
	Treatment	−0.094 (−0.151 ~ −0.032)	0.019	−0.094 (−0.151 ~ −0.032)	0.019		
	Work status	−0.072 (−0.139 ~ −0.003)	0.042	−0.072 (−0.139 ~ −0.003)	0.042		
	Income	0.081 (−0.003 ~ 0.145)	0.065	0.081 (−0.003 ~ 0.145)	0.065		
	Education	0.280 (0.206 ~ 0.357)	0.010	0.280 (0.206 ~ 0.357)	0.010		
Attitude	←						
	Knowledge	0.385 (0.330 ~ 0.491)	0.005	0.385 (0.330 ~ 0.491)	0.005		
	Life satisfaction	−0.228 (−0.301 ~ −0.143)	0.012	−0.175 (−0.250 ~ −0.096)	0.014	−0.052 (−0.086 ~ −0.031)	0.005
	Type of insurance	−0.058 (−0.118 ~ 0.009)	0.116	−0.058 (−0.118 ~ 0.009)	0.116		
	Education	0.207 (0.145 ~ 0.291)	0.005	0.099 (0.028 ~ 0.179)	0.008	0.108 (0.066 ~ 0.141)	0.014
	Health self-assessment	0.110 (0.015 ~ 0.197)	0.036	0.110 (0.015 ~ 0.197)	0.036		
	Weight loss and metabolic drugs					−0.042 (−0.066 ~ −0.021)	0.008
	Treatment					−0.036 (−0.064 ~ −0.015)	0.007
	Work status					−0.028 (−0.054 ~ −0.001)	0.038
	Income					0.031 (0.001 ~ 0.056)	0.046
	Age	−0.080 (−0.173 ~ 0.002)	0.056	−0.080 (−0.173 ~ 0.002)	0.056		
Practice	←						
	Attitude	0.211 (0.132 ~ 0.308)	0.012	0.211 (0.132 ~ 0.308)	0.012		
	Knowledge	0.389 (0.326 ~ 0.451)	0.010	0.308 (0.234 ~ 0.384)	0.005	0.081 (0.053 ~ 0.118)	0.006
	Disease	−0.136 (−0.182 ~ −0.066)	0.018	−0.136 (−0.182 ~ −0.066)	0.018		
	Treatment	−0.157 (−0.224 ~ −0.094)	0.015	−0.120 (−0.190 ~ −0.053)	0.012	−0.037 (−0.062 ~ −0.014)	0.012
	Work status	0.023 (−0.046 ~ 0.093)	0.562	0.051 (−0.012 ~ 0.109)	0.140	−0.028 (−0.052 ~ −0.001)	0.042
	Weight loss and metabolic drugs	−0.099 (−0.166 ~ −0.035)	0.012	−0.057 (−0.120 ~ 0.003)	0.057	−0.042 (−0.063 ~ −0.018)	0.012
	Health self-assessment	−0.061 (−0.121 ~ −0.063)	0.078	−0.084 (−0.148 ~ −0.017)	0.010	0.023 (0.004 ~ 0.047)	0.026
	Age	0.197 (0.126 ~ 0.250)	0.023	0.214 (0.149 ~ 0.267)	0.018	−0.017 (−0.044 ~ −0.001)	0.026
	Gender	0.135 (0.085 ~ 0.199)	0.004	0.135 (0.085 ~ 0.199)	0.004		
	Type of insurance					−0.012 (−0.030 ~ 0.001)	0.080
	Life satisfaction					−0.090 (−0.121 ~ −0.063)	0.009
	Income					0.031 (0.001 ~ −0.001)	0.043
	Education					0.130 (0.098 ~ 0.164)	0.009

CI, confidence interval.

In addition, knowledge (*β* (95% CI) = 0.385 (0.330 ~ 0.491), *p* = 0.005) and health self-assessment (β (95% CI) = 0.110 (0.015 ~ 0.197), *p* = 0.036) had direct effects on attitude. Taking weight loss and metabolic drugs (*β* (95% CI) = −0.042 (−0.066 ~ −0.021), *p* = 0.008), treatment (*β* (95% CI) = −0.036 (−0.064 ~ −0.015), *p* = 0.007), work status (*β* (95% CI) = −0.028 (−0.054 ~ −0.001), *p* = 0.038) and income (*β* (95% CI) = 0.031 (0.001 ~ 0.056), *p* = 0.046) had indirect effects on attitude. Furthermore, life satisfaction and education had effects on attitude including both direct (*β* (95% CI) = −0.175 (−0.250 ~ −0.096), *p* = 0.014), (*β* (95% CI) = 0.099 (0.028 ~ 0.179), *p* = 0.008) and indirect (*β* (95% CI) = −0.052 (−0.086 ~ −0.031), *p* = 0.005), (*β* (95% CI) = 0.108 (0.066 ~ 0.141), *p* = 0.014) part (Table 3).

Lastly, attitude (*β* (95% CI) = 0.211 (0.132 ~ 0.308), *p* = 0.012), gender (β (95% CI) = 0.135 (0.085 ~ 0.199), *p* = 0.004) and disease (*β* (95% CI) = −0.136 (−0.182 ~ −0.066), *p* = 0.018) all had direct effects on practice. Conversely, life satisfaction (*β* (95% CI) = −0.090 (−0.121 ~ −0.063), *p* = 0.009), taking weight loss and metabolic drugs (*β* (95% CI) = −0.042 (−0.063 ~ −0.018), *p* = 0.012), income (*β* (95% CI) = 0.031 (0.001 ~ −0.001), *p* = 0.043) and education (*β* (95% CI) = 0.130 (0.098 ~ 0.164), *p* = 0.009) had indirect effects on practice. Additionally, knowledge, treatment, health self-assessment, and age each demonstrated both direct and indirect effects on practice. Specifically, knowledge had a direct effect (*β* (95% CI) = 0.308 (0.234 ~ 0.384), *p* = 0.005) and an indirect effect (*β* (95% CI) = 0.081 (0.053 ~ 0.118), *p* = 0.006). Treatment had a direct effect (*β* (95% CI) = −0.120 (−0.190 ~ −0.053), *p* = 0.012) and an indirect effect (*β* (95% CI) = −0.037 (−0.062 ~ −0.014), *p* = 0.012). Health self-assessment had a direct effect (*β* (95% CI) = −0.084 (−0.148 ~ −0.017), *p* = 0.010) and an indirect effect (*β* (95% CI) = 0.023 (0.004 ~ 0.047), *p* = 0.026). Age had a direct effect (*β* (95% CI) = 0.214 (0.149 ~ 0.267), *p* = 0.018) and an indirect effect (*β* (95% CI) = −0.017 (−0.044 ~ −0.001), *p* = 0.026) on practice (Table 3).

## Discussion

The present study found a positive attitude, but moderate knowledge and practice level among patients with T2D and/or overweight/obesity regarding cardiovascular risk factor management. The results revealed several key findings that contribute to understanding this population’s approach to managing their cardiovascular health.

Firstly, the participants exhibited a positive attitude regarding cardiovascular risk factor management, which is encouraging. However, their knowledge and practice levels were only moderate. Likewise, a cross-sectional study examining KAP related to cardiovascular disease prevention among Chinese middle school students found that knowledge scores had the lowest pass rate, while attitudes toward health were generally positive (11). Additionally, a KAP study on hypertension conducted among patients in Lebanon revealed that while participants demonstrated moderate knowledge and practice, their attitudes were generally positive (20). Another KAP study focusing on lifestyle and cardiovascular risk factors in patients with metabolic syndrome reported that participants exhibited moderate levels of knowledge and practice, but showed high attitude scores toward CVD risk factors (21). This similarity indicates that both the healthy younger population and the high-risk older population may be aware of the importance of managing cardiovascular health, but they both lack the necessary information or motivation to implement effective management strategies. Our findings are consistent with previous studies conducted in populations at elevated cardiovascular risk. For example, Verma et al. reported moderate levels of knowledge and practice but a positive attitude toward cardiovascular disease prevention among patients with metabolic syndrome (13). Similarly, Siddique et al. found that older adults in rural Bangladesh demonstrated insufficient knowledge yet favorable attitudes (12). These similarities suggest that positive attitudes do not necessarily translate into optimal preventive practices, highlighting a common gap in adherence behaviors across high-risk populations. Consistently, a population-based study from Lebanon reported overall limited knowledge and practice despite fair attitudes toward cardiovascular diseases (22). This underscores the need for targeted education and intervention programs to bridge this gap and improve patients’ knowledge and practices.

Regarding specific knowledge areas, participants showed high awareness of the harms of poor lifestyle habits such as smoking and excessive alcohol consumption, as well as the benefits of moderate physical exercise. However, there was limited understanding of the benefits of healthy foods and the Mediterranean diet. A cross-sectional study conducted in India conducted a more thorough investigation into the dietary structure of patients with metabolic syndrome who also had high cardiovascular risk. The study found that 69% of participants agreed that they should reduce fat intake, while only 39% believed that their diet should include more vegetables and fruits. 41% of participants were willing to reduce their sugar intake, and only 18% were willing to reduce their salt intake (13). This highlights the need for targeted educational initiatives focusing on nutrition and dietary recommendations for cardiovascular health.

The practice dimension revealed areas of both strength and weakness. The high rate of smoking avoidance is commendable, indicating successful adherence to this aspect of cardiovascular health management. However, rates for consulting professionals to improve diet and lifestyle, regularly monitoring blood parameters, controlling dietary intake, exercising, actively seeking relevant information, regularly communicating with doctors, and participating in relevant educational guidance were low. This pattern aligns with broader evidence indicating that while awareness of healthy lifestyle behaviors such as regular exercise, balanced diet, and smoking cessation is common, actual adherence to these practices remains limited in both general and high-risk populations. Studies have shown that participation in lifestyle counseling, continuous health monitoring, and structured behavior modification programs is often inadequate, reflecting barriers in implementation and follow-up (23–26). A previous cross-sectional study made similar findings, with only 10.7 and 32% of participants demonstrating satisfactory practical behaviors in physical activity and dietary control, respectively (27). These findings underscore the need for interventions that promote and support these practices.

The correlation analysis revealed strong positive associations among knowledge, attitude, and practice, consistent with findings from earlier research (28–30), highlighting the interconnectedness of these components in cardiovascular risk factor management. This suggests that improving knowledge can positively influence attitude and practice, and vice versa.

The path analysis identified several factors that significantly influenced KAP scores. Notably, education had a positive direct effect on knowledge and attitude, indicating that higher educational levels may facilitate better understanding and more positive attitudes toward cardiovascular health management (22, 31). In addition, socioeconomic status, age, and gender also demonstrated differential effects on KAP outcomes in our study. For example, higher income was indirectly associated with better attitudes and practices, which may be attributed to greater access to healthcare resources and preventive services. Gender differences were observed in practice scores, with female participants showing better adherence to preventive behaviors, consistent with previous reports highlighting greater health awareness among women. Furthermore, age had both direct and indirect effects on practice, suggesting that older adults may have stronger motivation for disease prevention but may also face physical or resource-related barriers. These findings underscore the importance of tailoring interventions based on demographic characteristics to improve the effectiveness of cardiovascular disease prevention strategies (32). Income had a positive indirect effect on attitude and practice, suggesting that higher income levels may indirectly facilitate more positive attitudes and practices. These results are consistent with a prior cross-sectional study that identified significant links between variables like education level and monthly family income and elevated KAP scores related to cardiovascular health in older adults from rural Bangladesh (12), highlighting the need for tailored interventions aimed at preventing and managing cardiovascular diseases. On the other hand, disease status had a direct negative effect on practice, which aligns with a previous study’s finding that patients with lower waist circumference and lower fasting blood sugar exhibited significantly better KAP scores (13), indicating that the presence of T2D and/or overweight/obesity may impede the adoption of healthy practices.

Interestingly, health self-assessment had both a negative direct effect and a positive indirect effect on practice. This complex relationship suggests that while a positive self-assessment of health may indirectly promote healthy practices, it may also lead to a false sense of security that directly hinders the adoption of necessary preventive measures. Age had a positive direct effect and a negative indirect effect on practice, indicating that while older age may directly promote healthy practices, it may also indirectly hinder them through other factors (33).

However, there still exists limitations within this study. Firstly, the reliance on self-reported data may introduce bias, as participants may have under- or over-reported certain information, such as lifestyle habits, dietary intake, or medication adherence. Incorporation of objective measures, such as biometric data and medical records, might be able to complement self-reported information and reduce bias. Secondly, the study’s cross-sectional design restricts the ability to determine causal connections between variables. To gain a clearer understanding of how KAP evolve over time and influence the management of cardiovascular risk factors, longitudinal research is necessary. Lastly, this was a single-center study, and participants were recruited from specific locations, therefore the sample may not fully represent the broader population of patients with T2D and/or overweight/obesity across different regions and demographics. Conducting larger, multicenter studies involving more diverse populations could improve the generalizability of these results.

In conclusion, this study highlights the need for targeted interventions to improve knowledge, attitude, and practice regarding cardiovascular risk factor management among patients with T2D and/or overweight/obesity. Future research should focus on developing and evaluating the effectiveness of such interventions, particularly in areas such as nutrition education, self-efficacy enhancement, and promoting healthy practices.

## Data Availability

The original contributions presented in the study are included in the article/Supplementary material, further inquiries can be directed to the corresponding author.
