# Supplementary material for: Knowledge, attitude, and practice of cardiovascular disease prevention among patients with type 2 diabetes and/or overweight or obesity
Source: Front Public Health. 2025 Nov 27;13:1712899. doi: 10.3389/fpubh.2025.1712899 (PMC12695542; doi:10.3389/fpubh.2025.1712899)
Supplement: Supplementary file 1 [file Data_Sheet_1.docx]

**Supplementary Table 1.** Distribution of scores for knowledge dimension.

| **Knowledge** | **N (%)** | | |
| --- | --- | --- | --- |
|  | **Very familiar** | **Heard of it** | **Not clear** |
| 1. High cholesterol, high blood sugar, obesity, and high blood pressure are major risk factors related to cardiovascular diseases. | 293 (31.44) | 517 (55.47) | 122 (13.09) |
| 1. Healthy foods and the Mediterranean diet help control blood glucose, blood lipids, and blood pressure, reducing cardiovascular risk. | 216 (23.18) | 524 (56.22) | 192 (20.6) |
| 1. Moderate physical exercise and physical activity help control blood glucose, blood lipids, and blood pressure, maintaining cardiovascular health. | 393 (42.17) | 483 (51.82) | 56 (6.01) |
| 1. Poor lifestyle habits such as smoking and excessive alcohol consumption increase cardiovascular risk. | 400 (42.92) | 476 (51.07) | 56 (6.01) |
| 1. The capital city of China is Shanghai. |  |  |  |
| 1. Obesity increases the risk of cardiovascular disease in patients with Type 2 diabetes. | 301 (32.3) | 524 (56.22) | 107 (11.48) |
| 1. Weight loss can improve blood glucose control, blood lipid levels, and blood pressure in overweight and obese adults with Type 2 diabetes. | 279 (29.94) | 512 (54.94) | 141 (15.13) |
| 1. Proper blood pressure control can reduce the risk of cardiovascular events in diabetic patients. | 283 (30.36) | 518 (55.58) | 131 (14.06) |
| 1. Excessive alcohol consumption may lead to hypoglycemia and delayed hypoglycemia, weight gain, increased risk of high blood sugar and elevated blood pressure. | 289 (31.01) | 529 (56.76) | 114 (12.23) |
| 1. Smoking increases the risk of coronary heart disease, heart failure, peripheral artery disease, stroke, and cardiovascular death in patients with Type 2 diabetes. | 287 (30.79) | 510 (54.72) | 135 (14.48) |
| 1. Obesity combined with diabetes can exacerbate the occurrence and progression of chronic complications of diabetes. | 281 (30.15) | 529 (56.76) | 122 (13.09) |
| 1. For diabetic patients with obesity, it is important to manage weight alongside glycemic control to prevent diabetes complications and improve patients' quality of life. | 274 (29.4) | 534 (57.3) | 124 (13.3) |
| 1. For overweight or obese patients with Type 2 diabetes, psychological intervention, relaxation, and rational disease management are important for better disease control and improving quality of life. | 289 (31.01) | 538 (57.73) | 105 (11.27) |

**Supplementary Table 2.** Distribution of scores for attitude dimension.

| **Attitude** | **N (%)** | | | | |
| --- | --- | --- | --- | --- | --- |
|  | **Strongly agree** | **Agree** | **Neutral** | **Disagree** | **Strongly disagree** |
| 1. I believe that regular health check-ups (e.g., blood pressure, cholesterol, blood sugar) are important for managing Type 2 diabetes and overweight/obesity. | 315 (33.8) | 549 (58.91) | 51 (5.47) | 13 (1.39) | 4 (0.43) |
| 1. I am concerned about my cardiovascular health. | 123 (13.2) | 470 (50.43) | 227 (24.36) | 98 (10.52) | 14 (1.5) |
| 1. I believe that managing cardiovascular risk factors will have a positive impact on my life. | 276 (29.61) | 573 (61.48) | 59 (6.33) | 14 (1.5) | 10 (1.07) |
| 1. I believe I have sufficient ability and perseverance to manage cardiovascular risk factors. | 180 (19.31) | 537 (57.62) | 168 (18.03) | 40 (4.29) | 7 (0.75) |
| 1. I believe that managing cardiovascular risk factors will have a positive impact on my life. | 232 (24.89) | 605 (64.91) | 72 (7.73) | 20 (2.15) | 3 (0.32) |
| 1. I believe that controlling weight and dietary habits can reduce cardiovascular risk. | 268 (28.76) | 585 (62.77) | 59 (6.33) | 17 (1.82) | 3 (0.32) |
| 1. My family accompanies, supports, and supervises me in managing my health, controlling blood sugar, blood pressure, blood lipids, and weight. | 223 (23.93) | 581 (62.34) | 85 (9.12) | 39 (4.18) | 4 (0.43) |
| 1. Compared to lifestyle adjustments, I prefer to take medication. | 97 (10.41) | 376 (40.34) | 243 (26.07) | 194 (20.82) | 22 (2.36) |
| 1. Weight loss surgery carries certain risks, and I would not consider it lightly. | 232 (24.89) | 549 (58.91) | 88 (9.44) | 50 (5.36) | 13 (1.39) |
| 1. Managing cardiovascular risk factors requires long-term adherence rather than a quick fix. | 265 (28.43) | 600 (64.38) | 49 (5.26) | 13 (1.39) | 5 (0.54) |
| 1. Would you be willing to participate in lectures or popular science activities related to cardiovascular risk management? | 159 (17.06) | 530 (56.87) | 147 (15.77) | 90 (9.66) | 6 (0.64) |

P, positive; N, negative.

**Supplementary Table 3.** Distribution of scores for practice dimension.

| **Practice** | **N (%)** | | | | |
| --- | --- | --- | --- | --- | --- |
|  | **Always** | **Often** | **Sometimes** | **Rarely** | **Never** |
| 1. I regularly measure and monitor my blood pressure, blood sugar, and blood lipid levels. | 108 (11.59) | 363 (38.95) | 281 (30.15) | 134 (14.38) | 46 (4.94) |
| 1. I strictly control my dietary intake, such as limiting sugar, salt, and reducing high-fat food consumption. | 120 (12.88) | 386 (41.42) | 255 (27.36) | 113 (12.12) | 58 (6.22) |
| 1. I follow the doctor's prescribed medication regimen strictly. | 377 (40.45) | 329 (35.3) | 130 (13.95) | 53 (5.69) | 43 (4.61) |
| 1. I regularly engage in moderate-intensity physical exercise. | 151 (16.2) | 357 (38.3) | 237 (25.43) | 162 (17.38) | 25 (2.68) |
| 1. My recent smoking frequency. | 99 (10.62) | 132 (14.16) | 75 (8.05) | 75 (8.05) | 551 (59.12) |
| 1. My recent drinking frequency. | 34 (3.65) | 100 (10.73) | 146 (15.67) | 203 (21.78) | 449 (48.18) |
| 1. I consistently take my blood pressure, lipid-lowering, or blood sugar medications as prescribed. | 317 (34.01) | 237 (25.43) | 83 (8.91) | 82 (8.8) | 213 (22.85) |
| 1. I participate in physical exercise or other physical activities to maintain cardiovascular health. | 114 (12.23) | 255 (27.36) | 233 (25) | 171 (18.35) | 159 (17.06) |
| 1. I actively seek information and advice on managing cardiovascular risk factors related to diabetes/obesity. | 113 (12.12) | 397 (42.6) | 250 (26.82) | 123 (13.2) | 49 (5.26) |
| 1. I regularly visit the hospital for check-ups, cardiovascular assessments, and communicate with my doctor. | 104 (11.16) | 261 (28) | 301 (32.3) | 207 (22.21) | 59 (6.33) |
| 1. I have received education and guidance on managing cardiovascular risk factors through health education lectures or other means (P). | 97 (10.41) | 312 (33.48) | 270 (28.97) | 178 (19.1) | 75 (8.05) |
| 1. I have sought advice from a professional nutritionist, doctor, or other health expert to improve my diet and lifestyle. | 89 (9.55) | 268 (28.76) | 243 (26.07) | 230 (24.68) | 102 (10.94) |

P, positive; N, negative.

**Supplementary Table 4.** Model fit (first round).

| **Model Fit Indicators** | **Ref.** | **Measured results** |
| --- | --- | --- |
| **CMIN/DF** | 1-3 excellent, 3-5 good | 20.744 |
| **RMSEA** | <0.08 good | 0.146 |
| **IFI** | >0.8 good | 0.299 |
| **TLI** | >0.8 good | 0.014 |
| **CFI** | >0.8 good | 0.289 |

Ref., reference; CMIN/DF, Chi-square/Degrees of Freedom; RMSEA, Root Mean Square Error of Approximation; IFI, Incremental Fit Index; TLI, Tucker-Lewis Index; CFI, Comparative Fit Index.

**Supplementary Table 5.** Estimate of total effects (first round).

|  |  |  | **Estimate** | **S.E.** | **C.R.** | **P** |
| --- | --- | --- | --- | --- | --- | --- |
| **Knowledge** | **←** |  |  |  |  |  |
|  |  | **Age** | .019 | .010 | 1.805 | .071 |
|  |  | **Residence** | .180 | .158 | 1.138 | .255 |
|  |  | **Education** | 2.018 | .199 | 10.143 | *** |
|  |  | **Work status** | -.423 | .145 | -2.910 | .004 |
|  |  | **Income** | .496 | .174 | 2.846 | .004 |
|  |  | **Marital status** | -.256 | .269 | -.952 | .341 |
|  |  | **Disease** | -.134 | .136 | -.984 | .325 |
|  |  | **Treatment** | -.875 | .278 | -3.152 | .002 |
|  |  | **Weight loss and metabolic drugs** | -2.241 | .613 | -3.655 | *** |
|  |  | **Type of insurance** | .077 | .213 | .362 | .718 |
|  |  | **Health self-assessment** | .128 | .221 | .580 | .562 |
|  |  | **Life satisfaction** | -1.201 | .254 | -4.724 | *** |
| **Attitude** | **←** |  |  |  |  |  |
|  |  | **Knowledge** | .234 | .019 | 12.177 | *** |
|  |  | **Age** | -.016 | .006 | -2.570 | .010 |
|  |  | **Ethnicity** | .191 | .266 | .720 | .472 |
|  |  | **Education** | .405 | .124 | 3.269 | .001 |
|  |  | **Work status** | .101 | .086 | 1.170 | .242 |
|  |  | **Income** | .106 | .104 | 1.023 | .306 |
|  |  | **Marital status** | -.063 | .159 | -.396 | .692 |
|  |  | **Disease** | .025 | .081 | .315 | .752 |
|  |  | **Treatment** | -.269 | .165 | -1.630 | .103 |
|  |  | **Type of insurance** | -.248 | .126 | -1.973 | .049 |
|  |  | **Health self-assessment** | .514 | .131 | 3.922 | *** |
|  |  | **Life satisfaction** | -.907 | .152 | -5.956 | *** |
| **Practice** | **←** |  |  |  |  |  |
|  |  | **Attitude** | .410 | .064 | 6.376 | *** |
|  |  | **Knowledge** | .374 | .041 | 9.128 | *** |
|  |  | **Gender** | 2.012 | .413 | 4.867 | *** |
|  |  | **Age** | .075 | .012 | 6.200 | *** |
|  |  | **Residence** | -.595 | .184 | -3.224 | .001 |
|  |  | **Education** | .078 | .245 | .320 | .749 |
|  |  | **Work status** | .545 | .170 | 3.209 | .001 |
|  |  | **Income** | .196 | .204 | .963 | .336 |
|  |  | **Marital status** | .189 | .313 | .602 | .547 |
|  |  | **Disease** | -.783 | .159 | -4.930 | *** |
|  |  | **Treatment** | -1.382 | .325 | -4.250 | *** |
|  |  | **Weight loss and metabolic drugs** | -1.479 | .718 | -2.059 | .039 |
|  |  | **Health self-assessment** | -.401 | .260 | -1.544 | .123 |
|  |  | **Life satisfaction** | -.659 | .305 | -2.161 | .031 |

S.E., Standard Error; C.R., Confidence Ratio.

**Supplementary Table 6.** Model fit after removing paths with P > 0.05.

| **Model fit indicators** | **Ref.** | **Measured results**  **(Before adjustment)** | **Measured results**  **(After adjustment)** |
| --- | --- | --- | --- |
| **CMIN/DF** | 1-3 excellent, 3-5 good | 20.724 | 4.707 |
| **RMSEA** | < 0.08 good | 0.146 | 0.063 |
| **IFI** | > 0.8 good | 0.367 | 0.902 |
| **TLI** | > 0.8 good | 0.170 | 0.844 |
| **CFI** | > 0.8 good | 0.361 | 0.901 |

Ref., reference; CMIN/DF, Chi-square/Degrees of Freedom; RMSEA, Root Mean Square Error of Approximation; IFI, Incremental Fit Index; TLI, Tucker-Lewis Index; CFI, Comparative Fit Index.

**Supplementary Table 7.** Estimate of total effects after removing paths with P > 0.05.

|  |  |  | **Estimate** | **S.E.** | **C.R.** | **P** |
| --- | --- | --- | --- | --- | --- | --- |
| **Knowledge** | **←** |  |  |  |  |  |
|  |  | **Weight loss and metabolic drugs** | -2.222 | .614 | -3.617 | *** |
|  |  | **Life satisfaction** | -1.162 | .255 | -4.559 | *** |
|  |  | **Treatment** | -.873 | .278 | -3.138 | .002 |
|  |  | **Work status** | -.358 | .169 | -2.121 | .034 |
|  |  | **Income** | .472 | .190 | 2.483 | .013 |
|  |  | **Education** | 1.919 | .242 | 7.945 | *** |
| **Attitude** | **←** |  |  |  |  |  |
|  |  | **Knowledge** | .238 | .019 | 12.478 | *** |
|  |  | **Life satisfaction** | -.922 | .177 | -5.211 | *** |
|  |  | **Type of insurance** | -.254 | .133 | -1.914 | .056 |
|  |  | **Education** | .420 | .145 | 2.892 | .004 |
|  |  | **Health self-assessment** | .505 | .154 | 3.291 | .001 |
|  |  | **Age** | -.017 | .007 | -2.409 | .016 |
| **Practice** | **←** |  |  |  |  |  |
|  |  | **Attitude** | .430 | .064 | 6.764 | *** |
|  |  | **Knowledge** | .387 | .040 | 9.641 | *** |
|  |  | **Disease** | -.784 | .160 | -4.901 | *** |
|  |  | **Treatment** | -1.410 | .328 | -4.293 | *** |
|  |  | **Work status** | .318 | .183 | 1.732 | .083 |
|  |  | **Weight loss and metabolic drugs** | -1.483 | .723 | -2.050 | .040 |
|  |  | **Health self-assessment** | -.786 | .261 | -3.007 | .003 |
|  |  | **Age** | .094 | .013 | 7.275 | *** |
|  |  | **Gender** | 2.019 | .417 | 4.844 | *** |

S.E., Standard Error; C.R., Confidence Ratio.
